# A New Frontier in Cystic Fibrosis Pathophysiology: How and When Clock Genes Can Affect the Inflammatory/Immune Response in a Genetic Disease Model

**DOI:** 10.3390/cimb46090618

**Published:** 2024-09-18

**Authors:** Annalucia Carbone, Pamela Vitullo, Sante Di Gioia, Stefano Castellani, Massimo Conese

**Affiliations:** 1 Department of Clinical and Experimental Medicine, University of Foggia, 71122 Foggia, Italy; annalucia.carbone@unifg.it (A.C.); sante.digioia@unifg.it (S.D.G.); 2 Cystic Fibrosis Support Center, Ospedale “G. Tatarella”, 71042 Cerignola, Italy; pamelavitullo@gmail.com; 3 Department of Precision and Regenerative Medicine and Ionian Area (DiMePRe-J), University of Bari “Aldo Moro”, 70125 Bari, Italy; stefano.castellani@uniba.it

**Keywords:** cystic fibrosis lung disease, clock genes, circadian rhythms, immune-inflammation, ex vivo and in vivo models, REV-ERBα agonists, PGE2 and PGI2 analogs

## Abstract

Cystic fibrosis (CF) is a monogenic syndrome caused by variants in the CF Transmembrane Conductance Regulator (*CFTR*) gene, affecting various organ and systems, in particular the lung, pancreas, sweat glands, liver, gastrointestinal tract, vas deferens, and vascular system. While for some organs, e.g., the pancreas, a strict genotype-phenotype occurs, others, such as the lung, display a different pathophysiologic outcome in the presence of the same mutational asset, arguing for genetic and environmental modifiers influencing severity and clinical trajectory. *CFTR* variants trigger a pathophysiological cascade of events responsible for chronic inflammatory responses, many aspects of which, especially related to immunity, are not ascertained yet. Although clock genes expression and function are known modulators of the innate and adaptive immunity, their involvement in CF has been only observed in relation to sleep abnormalities. The aim of this review is to present current evidence on the clock genes role in immune-inflammatory responses at the lung level. While information on this topic is known in other chronic airway diseases (chronic obstructive pulmonary disease and asthma), CF lung disease (CFLD) is lacking in this knowledge. We will present the bidirectional effect between clock genes and inflammatory factors that could possibly be implicated in the CFLD. It must be stressed that besides sleep disturbance and its mechanisms, there are not studies directly addressing the exact nature of clock genes’ involvement in inflammation and immunity in CF, pointing out the directions of new and deepened studies in this monogenic affection. Importantly, clock genes have been found to be druggable by means of genetic tools or pharmacological agents, and this could have therapeutic implications in CFLD.

## 1. Introduction

**Figure 1:**
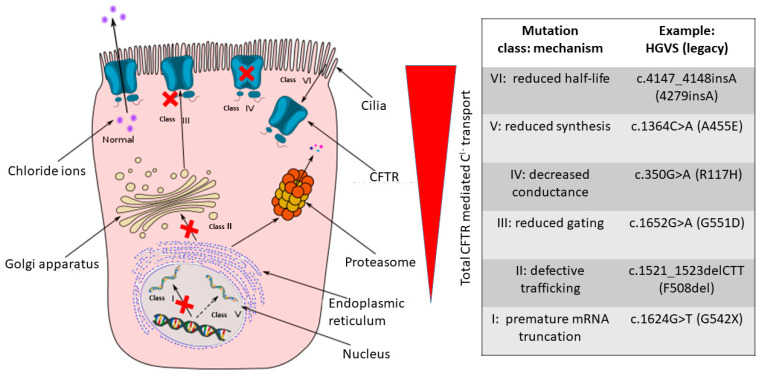
CFTR mutation classes, mechanisms, and principal examples. CFTR variants are reported as HGVS nomenclature (legacy). The left part has been adapted from Wikimedia Commons [16]. While class I mutations are associated with the annihilation of CFTR-mediated chloride transport, chloride transport gradually increases through the remaining five classes with the greatest activity being observed in class IV–VI mutations [17]. The examples are reported as HGVS (Human Genome Variation Society) and legacy nomenclatures. The dashed arrow indicates reduced CFTR synthesis in class V mutations. Red crosses identify mutation classes where the CFTR synthesis, processing or gating is abolished or reduced. The red triangle depicts the decrease in total CFTR mediated chloride transport and is referred to the right part of the Figure (i.e., class mutations).

Cystic fibrosis (CF) (OMIM 219700) is a monogenic disorder, which is directly caused by over 2500 gene variants in the CF Transmembrane Conductance Regulator (*CFTR*) gene (OMIM, 602421) [1]. This allelic heterogeneity undermines the possibility of knowing the effect of *CFTR* gene variants on the clinical phenotype [2]; indeed, 20–30% of them are lacking an associated phenotype [3,4,5,6]. Given this drawback, pathogenic CFTR variants are classified into six mutation classes based on the mechanisms leading to CFTR protein lack or dysfunction at the appropriate subcellular location, i.e., the apical membrane of epithelial cells (Figure 1). Not all classifications are perfect: indeed, *F508del* (legacy nomenclature), the most frequent variant in Caucasian people (approximately 70% of the CF alleles) [5], not only is hampered in its trafficking to the plasma membrane and degraded (class II), but also the few molecules that reach their target are dysfunctional (class III) [7]. Moreover, class I variants, exiting in no production of the CFTR protein, are determined by different molecular mechanisms, i.e., nonsense, frameshift and some splice variants. Thus, others have sometimes broken these variants in two classes, class I remaining with “no protein”, and class VII being “no mRNA” [8,9].

While a strict genotype-phenotype correlation has been observed for the pancreas, the lung disease varies between individuals with CF in the presence of the same mutational asset. Environmental modifiers, such as secondhand tobacco smoke exposure, allergens, socioeconomic status, health care access, and air pollution, may modify the onset/severity of lung disease [10,11,12]. In addition, also other CFTR variants in *cis* [13] and other loci outside that of *CFTR* have been implied in contributing to this phenotype variability. Genome-wide association studies have allowed to find polymorphisms associated with various CF-associated conditions, such as lung disease, meconium ileus, diabetes, and liver disease [6]. Also inflammatory gene variants are implied in the modification of the clinical outcome of the CF respiratory disease [14]. The allelic heterogeneity, intertwined with modifiers genes, has entailed a bunch of etiologic therapies, encompassing those based on the modifications of DNA (gene editing or gene adding/replacement), RNA (splice modulation, nonsense-mediated decay inhibition, readthrough), and proteins (modulators) [15]. However, CF-associated immune-inflammation disorder is an orphan yet. An agnostic mutation approach would permit treating all CF patients at once.

CF is a syndrome, whose pathological hallmark is the derangement of ion and fluid homeostasis at the level of secreting/adsorptive epithelia of various organs, including the respiratory tract, the gastrointestinal tract, sweat glands and the vas deferens. Hyperinflammatory response is the culprit for all the pathophysiological consequences stemming from the CFTR protein dysfunction or lack. From this viewpoint, CF is an orphan disease, missing efficacious anti-inflammatory therapies that can limit the destructive process at the level of the various affected organs and systems and leading to fibrosis.

The aim of this review is to discuss current evidence on the clock genes role in immune-inflammatory responses at the lung level. While information on this topic is known in other chronic airway diseases (chronic obstructive pulmonary disease and asthma), CF lung disease (CFLD) is lacking in this knowledge. We will present the bidirectional effect between clock genes and inflammatory factors that could possibly be implicated in the CFLD. It must be stressed that besides sleep disturbance and its mechanisms, there are not studies directly addressing the exact nature of clock genes’ involvement in inflammation and immunity in CF, pointing out the directions of new and deepened studies in this monogenic affection. Finally, we will give a glimpse of this new frontier in CF treatment by exploring the druggability of clock genes.

### Cystic Fibrosis Lung Disease

In people with CF (PWCF), lung disease is the major determinant of morbidity and mortality [18,19,20]. CFLD is characterized by opportunistic microorganism colonization and infection (e.g., *Pseudomonas aeruginosa*, *Burkholderia* spp., *Achromobacter* spp., *Stenotrophomonas maltophilia*, anaerobes, nontuberculous mycobacteria, and fungi) [21]. Moreover, the progression of CFLD is also influenced by infection with respiratory viruses, such as influenza, which is linked to the greatest decreases in lung function [22]. Chronic inflammation then ensues, involving epithelial cells, innate immune cells (macrophages and neutrophils) and adaptive immune cells (various types of T cells) [23,24,25]. CFLD is basically a mucosal immunodeficiency disease whose hallmarks are epithelial innate immune dysfunction, oxidative stress, alterations in resident and recruited immune cells, and remodeling of the airways [26,27]. CFLD is derived from alterations in inflammatory responses that drive the damage of airway structures, namely collagen and elastin, leading to bronchiectasis and producing a vicious cycle between inflammation and infection [28].

Airway epithelial cells (AECs) with tight junctions, surfactant film and mucociliary activity together with dendritic cells (DCs) and macrophages constitute a structural and functional barrier for the respiratory system against harmful pathogens [29].

CFTR dysfunction determines the loss of epithelial barrier function obliterating endotoxin tolerance, where microbial breach of the epithelial barrier orchestrates elevated pattern recognition receptor (PRR)-mediated signaling followed by leukocyte infiltration in the parenchyma or even sepsis when microbes enter the circulation [30]. This pattern-associated molecular pattern (PAMP)-mediated hyperactivation leads to exaggerated inflammasome activation due to nuclear factor (NF)-κB (NF-κB) and redox imbalance [29,31,32] with a heightened release of pro-inflammatory cytokines. The development of a pro-inflammatory state in CF epithelial cells and lymphocytes is also due to the reduced production and nuclear localization of peroxisome proliferator-activated receptors (PPARs), transcription factors that normally counteract the action of NF-κB, which is activated by the hyperproduction of reactive oxygen species (ROS) [33,34,35,36]. The induction of a systemic inflammatory response [24] is thus initiated and aggravated not only by the loss of epithelial integrity but also by a vascular dysfunction. In the context of systemic inflammation occurring in CF, it is worth noting that a dysfunctional endothelium might be the result of a dual involvement. One is intrinsic and explained by the dysfunctional CFTR leading to heightened IL-1β production and upregulated endothelial adhesion molecules VCAM-1, ICAM-1, P-selectin and E-selectin [37,38,39,40], which were found inversely related to disease severity and may reflect a state of chronic inflammation (see other references in [41]). The other is extrinsic, given the high levels of circulating mediators, such as TNF-α, IL-1β, IL-2, IL-6, IL-7, IL-8, IL-17, VEGF, and CD40 ligand [42,43,44,45,46,47,48], inducing in turn pro-inflammatory changes in endothelial cells [49].

CF inflammatory and immune cells share the same perturbations of cellular processes affecting epithelial and endothelial cells, namely cellular integrity, autophagy, cellular metabolism, oxidative burst, protein chlorination, pathogen clearance, endotoxin tolerance, and cell death, among others [41]. Thus, in leukocytes with CFTR dysfunction (especially neutrophils and macrophages), the loss of CFTR function results in reduced lysosomal acidification and Cl influx into phagosomes hindering the production of reactive chloride species (RCS) with consequent reduced pathogen clearance [50,51,52,53,54,55,56,57]. Autophagy, playing a role in eliminating intracellular pathogens, is also defective in CF macrophages contributing to the defective clearance of *B. cenocepacia* [58,59,60]. Other dysfunctions of macrophages and neutrophils in CF concern a dysregulated inflammatory phenotype and lipid disorders, which have been reviewed elsewhere [50,61]. Reduced mitochondrial ROS production by CF *F508del*/*F508del* murine macrophages has been also invoked for the defective bacterial killing with respect to *B. cenocepacia* infection [62].

## 2. Physiology and Pathophysiology of Clock Genes in Inflammation and Immunity

Circadian rhythms refer to the physical, mental, and behavioral fluctuations that organisms undergo within a 24 h cycle. While light and darkness play the most significant role in regulating these rhythms, they are also influenced by factors such as food consumption, stress levels, physical activity, social interactions, and environmental temperature. These rhythms are present in most living beings, including animals, plants, and microorganisms [63]. In humans, almost every tissue and organ has its own circadian rhythm, which is synchronized with the daily cycle of day and night. These rhythms impact key bodily functions, including sleep patterns, hormone release, appetite, digestion, and body temperature. The system responsible for regulating an organism’s internal sense of time and controlling circadian rhythms is known as the biological clock. This system consists of proteins, encoded by thousands of genes, that switch on and off in a precise sequence. A master clock orchestrates the coordination of all the biological clocks within the organism [64]. In vertebrates, including humans, the master clock is located in the brain. In humans, this master clock consists of a large cluster of nerve cells that make up the structure known as the suprachiasmatic nucleus (SCN). One of the SCN’s key functions is regulating the production of the hormone melatonin, based on the amount of light detected by the eyes. As evening approaches, the master clock signals the brain to increase melatonin production, inducing sleepiness. Additionally, the SCN ensures that circadian rhythms in various tissues and organs throughout the body remain synchronized [65].

In mammals, the core circadian clock operates through a complex system of interconnected transcription factors, which form multiple transcription-translation feedback loops. These loops activate and inhibit each other, generating 24 h rhythms at the molecular level [66]. This network is often referred to as the core clock and consists of the transcription factors BMAL1 [brain and muscle aryl hydrocarbon receptor nuclear translocator (ARNT)-like 1; encoded by *Arntl*], CLOCK (circadian locomotor output cycles kaput), PER1/2/3 (period circadian protein homologs), and CRY1/2 (cryptochromes 1/2) [66,67]. At the protein level, BMAL1 and CLOCK combine to form a heterodimeric transcription factor. This complex binds to the enhancer (E)-box regions of target genes, including those within the gene loci of the negative regulators Per1/2/3 and Cry1/2, thereby triggering their expression [66]. Once expressed, PER and CRY also form a heterodimeric transcription factor complex that inhibits the expression of BMAL1 and CLOCK until concentrations of the repressors are low enough to restart the cycle [66,67,68]. Additionally, the REV-ERB nuclear receptor α/β [nuclear receptor subfamily 1 group D (NR1D); encoded by *Nr1d1/2*] and the retinoic acid receptor (RAR)-related orphan receptors (RORs) form a secondary feedback loop that stabilizes the core molecular clock [66,67].

A more focused presentation of circadian clock involvement in epithelial and leukocyte physiology will follow. For instance, the circadian clock regulates immune cell numbers, migration, and function. In mammals, the number of circulating leukocytes fluctuates throughout the day, reaching its highest point during the rest phase in nocturnal mice [69] and diurnal humans [70]. Circadian clocks also modulate leukocyte migration across the body, effectively gating the number of leukocytes at specific sites throughout the day [69,71,72,73]. Leukocyte REV-ERBα regulates the rhythmic infiltration of neutrophils into the lungs following lipopolysaccharide (LPS) exposure [74]. Moreover, leukocyte effector functions, along with the ability of activated immune cells to proliferate and produce cytokines, are influenced by the time of day [75,76,77,78]. Most circadian studies in the immune system have focused on the role of BMAL1, because it is the only clock gene whose sole absence abrogates rhythmicity [68]. Deficiencies in various clock components, such as *Bmal1* alone, or double knockouts of *Cry1* and *Cry2*, or *Per1* and *Per2*, have been linked to altered inflammatory responses, underscoring the critical importance of the circadian clock in maintaining proper immune function [67]. For example, in *Bmal1*-deficient mice, the circadian rhythm of granulocyte oscillation in the lungs after LPS stimulation is entirely absent [79]. Removing *BMAL1* expression in bronchiolar epithelial club cells ablated their diurnal secretion of C-X-C motif chemokine ligand 5 (CXCL5), which is a neutrophil chemoattractant, thus leading to increased CXCL5, C-C Motif Chemokine Ligand 20 (CCL20), CCL8 levels and aggravated neutrophilic inflammation in response to LPS treatment [80]. Bronchiolar epithelial cells require BMAL1 expression to respond to oscillating glucocorticoid (GC) levels, and their rhythmic production of CXCL5 is dependent on the cell-intrinsic expression of the GC receptor (GR) [81]. Many aspects of airway epithelial cell biology, including mucus production by goblet cells, ion transport, fluid and pH regulation, as well as cell differentiation and turn-over, are still to be disclosed in their relation with clock genes function and/or dysfunction [82]. Additionally, BMAL1 plays a crucial role in regulating macrophage morphology, motility, metabolism, and antibacterial activity [83,84,85,86].

Recent studies have focused on the connections between clock components, particularly REV-ERBα, and inflammasome pathways [87,88,89,90]. In mouse and human macrophages, NLR family pyrin domain containing 3 (NLRP3, a major component of the inflammasome) is expressed in a diurnal manner under the control of REV-ERBα [88]. The pharmacological activation of REV-ERBα inhibited the NLRP3 inflammasome and alleviated the severity of experimental colitis [88,91]. Furthermore, REV-ERBα was shown to suppress the NF-κB/NLRP3 axis, with rev-erbα-deficient mice displaying increased NLRP3 inflammasome activity [91]. These findings suggest that REV-ERBα plays a significant role as a mediator in the clock machinery’s anti-inflammatory functions.

At the molecular level, some signal transduction pathways have been implicated in the modulation that clock genes expression can exert on inflammation and vice versa [92,93]. On one hand, clock proteins impact gene transcription both directly and by recruiting activating or repressing enzymes to the promoter regions of immune-associated genes. On the other hand, they exert their pro- or anti-inflammatory role physically interacting with inflammatory factors. For example, the pro-inflammatory action of CLOCK is mediated by direct interaction with components of the NF-κB pathway [94] but also via transactivation of the chemotactic cytokines *Ccl2*, *Ccl8*, and *S100a8* genes via complexing with BMAL1 [95]. In addition, PER1 mediates its repressive effects on macrophage inflammatory response through the binding with PPARγ [96]. Conversely, inflammation and immunity can influence clock genes. NF-κB, a master regulator of immune and inflammatory responses, inhibits clock repressors, including *Per*, *Cry*, and *Rev* genes, both in the unstimulated state and in response to inflammatory challenges [97]. Overall, a key mechanism in the interrelationship between clock proteins and the inflammatory pathways is the NF-κB pathway.

The disruption of clock gene regulation during chronic lung diseases plays an essential role in augmented oxidative stress, inflammatory response, metabolic imbalances, hypoxia/hyperoxia, mucus secretion, dysregulated autophagy, and alters pulmonary function [98,99]. Clock gene pathways have been found dysregulated in a lung disease, i.e., chronic pulmonary obstructive disease (COPD); in particular, it was found that the circadian clock signaling pathways were downregulated in COPD patients and also by cigarette smoking exposure in mice. The daily variation in exacerbation frequency and occurrence in COPD and asthma is influenced by the circadian timing system driving changes in airway caliber, airway resistance, respiratory symptoms, mucus hypersecretion and immune-inflammatory responses [98].

## 3. Role of Clock Genes in Cystic Fibrosis

Evidence suggests that PWCF may have intrinsic circadian disturbances resulting in dysfunctional gene regulations that may affect the circadian clock and, ultimately, sleep quality and lung function [100,101,102,103]. Although PWCF may be inherently prone to lack of sleep from chronic coughing, hypoxemia, hypercapnia, gastrointestinal reflux, abdominal pain, and medications [104,105,106,107], CFTR dysfunction has been implicated in rhythm and sleep disturbances in CF mice [108]. Thus, expression of the circadian clock genes was examined in multiple tissues of both CF and wild-type (WT) mice under two sleep conditions. CF mice homozygous for the *F508del* CFTR variant and control mice were allowed to sleep undisturbed or were kept awake for 6 h prior to sacrifice. At baseline, and in comparison with WT mice, CF mice had increased expression of *Clock* in the brain and jejunum, increased expression of *Bmal1* in the jejunum, and increased expression of *Cry2* in the brain. In the sleep-deprived state, the CF group showed an increased expression of *Clock*, *Bmal1*, and *Per2* in the brain as well as of *Per1* and *Per2* in the lung compared to the non-CF group. On the other hand, *Cry1* and *Cry2* expressions were decreased in the adipose tissue as well as *Cry1* in the colon of CF mice. A comparison between these groups revealed increases in expression in the lungs of all measured gene transcripts apart from *Cry1* in CF mice that had been sleep deprived. In all, these data suggest that CFTR dysfunction may cause dysregulation in the expression of some circadian clock genes which is exacerbated by sleep deprivation. Whether it is possible that these alterations in clock genes, especially at the lung level, may contribute to sleep disturbances, pulmonary phenotype, immune dysregulation or infection susceptibility, all these issues were not investigated. Furthermore, the limit of this study was that it considered CF mice, which are distant in the lung physiology and pathophysiology of humans. Thus, further studies on CF animal models closest to human pathophysiology are needed. Finally, a demonstration that CFTR dysfunction was causative of this dysregulation was not formally asserted; since adult mice were the object of this study, it could be that this dysregulation occurred with time and independently of *CFTR* mutation. Further studies employing mice at different ages, mirroring the chronic progression of CF lung disease, are needed to understand this issue.

In the attempt to understand the mechanism(s) underpinning the circadian clock gene dysregulation in CF mice, Barbato and colleagues investigated the role of histone deacetylase 6 (*Hdac6*) [109], which is a microtubule-modifying protein. This work stems from previous research showing that CF cells and CF mice show reduced tubulin acetylation and an altered microtubule polymerization with multiple consequences including altered endosomal trafficking, increased perinuclear cholesterol accumulation and inflammatory NF-κB signaling [110,111,112,113,114], which were reversed by an inhibition of Hdac6 [110,113]. In the CF mouse model, *Hdac6* knocking-out expression or its pharmacological inhibition resulted in the reduction in inflammatory responses and increased bacterial clearance rates of mice challenged with *P. aeruginosa* airway infection [115,116] as well as in the reversion of reduced growth and depression-like behaviors [117,118]. Importantly, mice lacking expression of the microtubule-regulating protein tubulin polymerization promoting protein (*Tppp*) exhibit phase-shifted sleep/activity cycles, altered circadian activity rhythms, a parallel altered clock gene expression profile, and reduced serum melatonin concentration [119], recapitulating some alterations found in CF mice. To note, knocking down *Tppp* expression recapitulates CF-like cellular phenotypes in airway epithelial cells [113], which is a significant finding, since *Tppp* is located in a region of chromosome 5 containing modifiers of CF airway disease severity [120].

In the study by Barbato et al. [109], to experience sleep disturbance, mice were subjected to 2 weeks in 12:12 light-dark (LD) conditions followed by 2 weeks in dark-dark (DD) conditions. Four mouse models were used: a WT control, a *F508del* CF mouse model, *Tppp*^−/−^ mice to model microtubule instability, and a CF model crossed with an *Hdac6*^−/−^ mouse line as a means to correct the microtubule issues in CF (CF/*Hdac6*). Locomotor activity (LA) refers to the movement from one location to another during both photoperiods and is computed in hours. Typical measures used in the analysis of circadian rhythmicity are ‘acrophase’ ‘trough’, and ‘activity onset’. Acrophase is the time at which the peak of the circadian rhythm occurs; a trough refers to a period or point in time when the level of physical activity (movement, such as walking or running) is at its lowest; activity onset is the time that animals start being active, and in the case of rodents, it typically refers to the time around the lights-off phase [121]. Zeitgeber time (ZT) is used as a reference in LD; lights on = ZT0 and lights off = ZT12. LA characteristics of CF and *Tppp*^−/−^ mice showed numerous differences throughout both photoperiods when compared with WT. On the other hand, locomotor activity in CF/*Hdac6* mice was not different from WT mice, but it was significantly elevated when compared with CF and *Tppp*^−/−^ mice. The DD periodicity was significantly longer in the CF (24.67 h) and *Tppp*^−/−^ (24.63 h) groups compared with WT controls (23.83 h) (*p* = 0.01 and *p* = 0.05, respectively). Moreover, all the other timing of LA characteristics (LD acrophase, LD trough, and LD activity onset) were altered in CF and *Tppp*^−/−^ mice and recovered in CF/*Hdac6* mice. In addition, significant differences in mean activity per hour in both LD and DD photoperiods were noted in CF and *Tppp*^−/−^ mice as compared to WT mice, while no differences were found between the WT and CF/*Hdac6* groups for either parameter. The gene expression of clock genes was studied in the SCN, and it was appreciated that the deletion of *Tppp* causes clock gene expression changes that mimic CF, which are reverted to WT patterns by the deletion of *Hdac6*.

**Figure 2:**
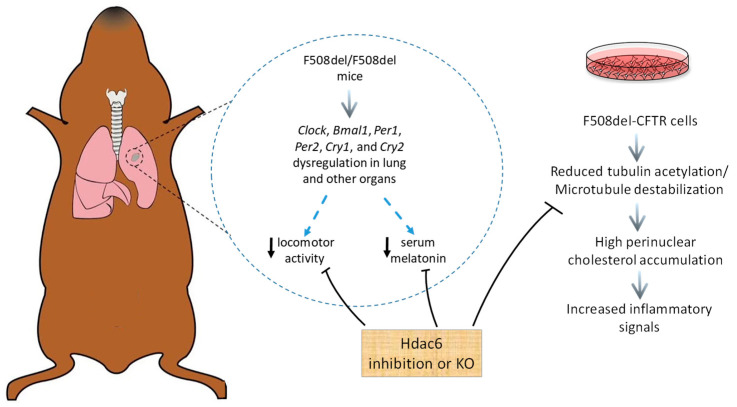
Alteration of circadian clock genes and derived phenotypes in CF mice and cells. CF (*F508del*/*F508del*) mice show dysregulation of clock genes expression in lungs and other organs (brain, colon, fat, jejunum, and skeletal muscle tissues). Due to a still-unknown mechanism (denoted by blue dashed arrows), reductions in locomotor activities, phase shift, and decreased serum melatonin levels occur. On the other hand, CF cells in cultures are denoted by microtubule destabilization with further consequences, such as a high perinuclear accumulation of cholesterol and increased pro-inflammatory signaling. Hdac6 KO or inhibition relieves all these phenotypes in vivo and in vitro, suggesting that microtubule dynamics are a modulator of circadian rhythm regulation.

At all the time points considered (ZT6, ZT12, ZT18, ZT0), serum melatonin levels were significantly lower in CF and *Tppp*^−/−^ mice than WT mice. Overall, CF mice do exhibit clear changes in circadian rhythm (CR) regulation encompassing sporadic and reduced overall locomotor activity, phase shift in activity onset, altered timing of CR-related gene expression, and reduced melatonin expression. Moreover, CF-related CR phenotypes are replicated by the mouse model of microtubule instability, *Tppp*^−/−^ mice, and reversed to WT profiles by the depletion of the tubulin deacetylase Hdac6 expression (Figure 2). However, and most importantly to this review, the relationship between circadian regulation and inflammation needs to be determined. Whether the presence of chronic inflammation in CF is altering CR regulation or if the lack of melatonin production and CR phase shifts in CF are impacting inflammatory responses needs to be addressed in future studies.

In addition to sleep disturbance and its mechanisms, there are not studies directly addressing the exact nature of clock genes’ involvement in inflammation and immunity in CF. However, to better frame the relevance of clock genes and circadian rhythm in CF inflammatory and infection events, we can recall that peripheral tissues and their cells, including airway epithelial cells, possess an intrinsic circadian clock responsible for rhythmic immune oscillations with the time of day [80,122,123]. Through an LPS-infection lung model, it was demonstrated that nonciliated club cells control circadian variations in bronchoalveolar lavage fluid (BAL) neutrophils counts via the secretion of the CXCL5 chemokine. Importantly, these variations were lost with the genetic ablation of *Bmal1*, resulting in an exaggerated inflammatory response [80]. In a subsequent work, the same group assessed the lung responses in mice bearing the club cell deletion of *bmal1* (CCSP-*Bmal1*^−/−^) showing BAL neutrophilia, heightened cytokine and chemokine levels, altered biomechanical function, and airway-centric fibrosis under basal and chronic influenza virus infection [124]. In the airway epithelium, club cells are nonciliated, nonmucous, secretory cells, whose main secreted protein is club cell secretory protein (CCSP), which is involved in reducing airway inflammation in response to oxidative stress and infections [125]. Recently, serum CCSP deficit has been found to be associated with lung disease severity in children with CF [126]. Laser-captured club cells, investigated at different times of day, also showed a reprogramming of the transcriptome ensuing in the disruption in genes of the core circadian clock and pathways regulating cell metabolism (lipids and lipid proteins), extracellular matrix, and chemokine signaling but also the gain of a novel rhythmic transcriptome in targeted cells. In an in vitro-airway liquid interface (ALI) model obtained culturing primary tracheal epithelial cells derived from CCSP-*Bmal1*^−/−^ mice and wild-type (WT) littermate mice, they observed similar increases of chemokines (*Cxcl5* and *Cxcl15*) that were appreciated in vivo, indicating that dysregulation of the chemokine pathways operates in a cell-intrinsic manner upon *Bmal1* deletion [124]. Some other authors that have used global *Bmal1* knockout models have described broadly similar pulmonary phenotypes, including an elevated pulmonary neutrophilia [79], aberrant responses to chronic inflammatory stimuli [127,128], and impaired mechanical function [129].

Another in vivo study emphasized the role of the rhythmic circadian repressor REV-ERBs in the coupling of the pulmonary clock to innate immunity. REV-ERBα knockout mice showed an exaggerated neutrophilic inflammation, accompanied by augmented chemokine and cytokine responses, including CXCL5, the chemokine required for the BMAL1 effect, both upon LPS challenge and also in unchallenged mice at steady state [74]. Overall, these data suggest the pivotal role of REV-ERB proteins as homeostatic anti-inflammatory controllers in the lung. At this point, it must be stressed that these in vivo studies should be performed in CF mice or other CF animal models to figure out whether *Cftr* expression and/or function is involved in pathological lung disease and which mechanisms are at work. Further hints should be derived from the evaluation of other immune cells in the lungs in the CF context, such as macrophages, and how they respond to respiratory tract infection according to circadian rhythms [130].

## 4. Discussion and Future Outlook

In the context of cystic fibrosis, new frontiers in the regulation of clock genes offer promising prospects for a more in-depth and potentially therapeutic understanding of the disease. Clock genes, which orchestrate circadian rhythms and influence various biological processes, are emerging as crucial elements in modulating the inflammatory response and epithelial function, which are two key aspects of cystic fibrosis. Although a direct proof of clock genes modulating the CF hyperinflammatory status in the lung is missing, dysfunctions in clock genes may ultimately contribute to the progression of the disease, influencing the production of mucus and the activity of ion channels, such as CFTR. For example, an alteration of circadian rhythms occurring in chronic airway diseases can influence mucus secretion and composition [98,131,132], while abnormalities in clock genes can exacerbate inflammation and compromise the immune response [98,99,133]. Although an involvement of CFTR has been invoked in the physiology of SCN neurons and circadian rhythm dysregulation [103], no studies have highlighted a direct influence of clock genes on the regulation of CFTR function and the ion homeostasis of airway epithelial cells as well as their impact on the inflammatory response; thus, these relationships should be investigated.

If we overcome these knowledge gaps, the manipulation of circadian rhythms through the use of targeted drugs or interventions based on clock gene modulators could represent an innovative therapeutic strategy to improve symptoms and slow the progression of CF lung disease. Potential approaches include the use of clock gene agonists or antagonists to restore a normal circadian rhythm, which could improve the effectiveness of existing treatments and contribute to a better quality of life for patients. Previous studies have shown the strong anti-inflammatory functions of REV-ERBα, in addition to its role in the circadian clock [92]. On the basis of this important anti-inflammatory role, REV-ERBα is currently used as a target in therapeutic studies. Rats with mechanical ventilation-induce lung injury had a better recovery and showed decreased TNF-α lung tissue levels when treated with the synthetic agonist SR9009 [134]. In vitro analyses demonstrated that REV-ERBα agonists SR8278 and GSK1362 reduced *Il6*, *Ccl2*, *Cxcl1*, and *Cxcl2* expression in LPS-treated alveolar macrophages as well as *Cxcl5* in IL-1β-stimulated bronchial epithelial cells [74]. Pre-treatment with the GSK4112 agonist reduced cigarette smoke extract (CSE)/LPS induced pro-inflammatory cytokines release from both human primary small airway epithelial cells derived from COPD patients and mouse lung fibroblasts [135]. REV-ERBα agonist SR9009 reduced acute cigarette smoke-induced inflammatory response and abnormal epithelial-mesenchymal transition (EMT) in the lungs, whereas GSK4112 inhibited TGF-β/cigarette smoke-induced fibroblast differentiation in human fetal lung fibroblasts [136].

Another study found that GSK4112 can inhibit the expression of NLRP3 and reduce the production of IL-1β in an LPS-induced inflammation model [90]. It was also shown that an NF-κB-driven long noncoding RNA (lncRNA), Lnc-UC, can promote the expression of REV-ERBα and, in a negative feedback loop, can inactivate NF-κB and the NLRP3 inflammasome in macrophages [137]. The circadian clock signaling pathways were found to be downregulated in COPD patients and by CS exposure in mice and restored by the ex vivo treatment of lung organoids with GSK4112. Importantly, in the same study, lung organoid formation was restored in the presence of 5% CSE also by in vivo treatment with the PGE2 agonist misoprostol and the PGI2 agonist iloprost [138]; this last drug was already clinically employed in patients with pulmonary hypertension [139].

Also, the microtubule dysfunction in CF may be the target of intervention. Although histone deacetylase (HDAC) inhibition controls *P. aeruginosa*-LPS-induced airway inflammation and CF-lung disease [116], the identification of specific HDACs that control CFTR processing and lung disease would lead to the development of selective HDAC-inhibitor drugs.

Overall, these studies indicate that the clock gene dysfunction is druggable and hold promise for therapeutic strategies hitting PGE2 signaling pathways or HDAC. Future research will need to delve deeper into the exact mechanism by which clock genes influence CF lung pathophysiology and evaluate the effectiveness of novel clinical interventions targeting these genetic modulators, especially in those PWCF with genotypes not currently amenable to the pharmacological correction of CFTR function.
